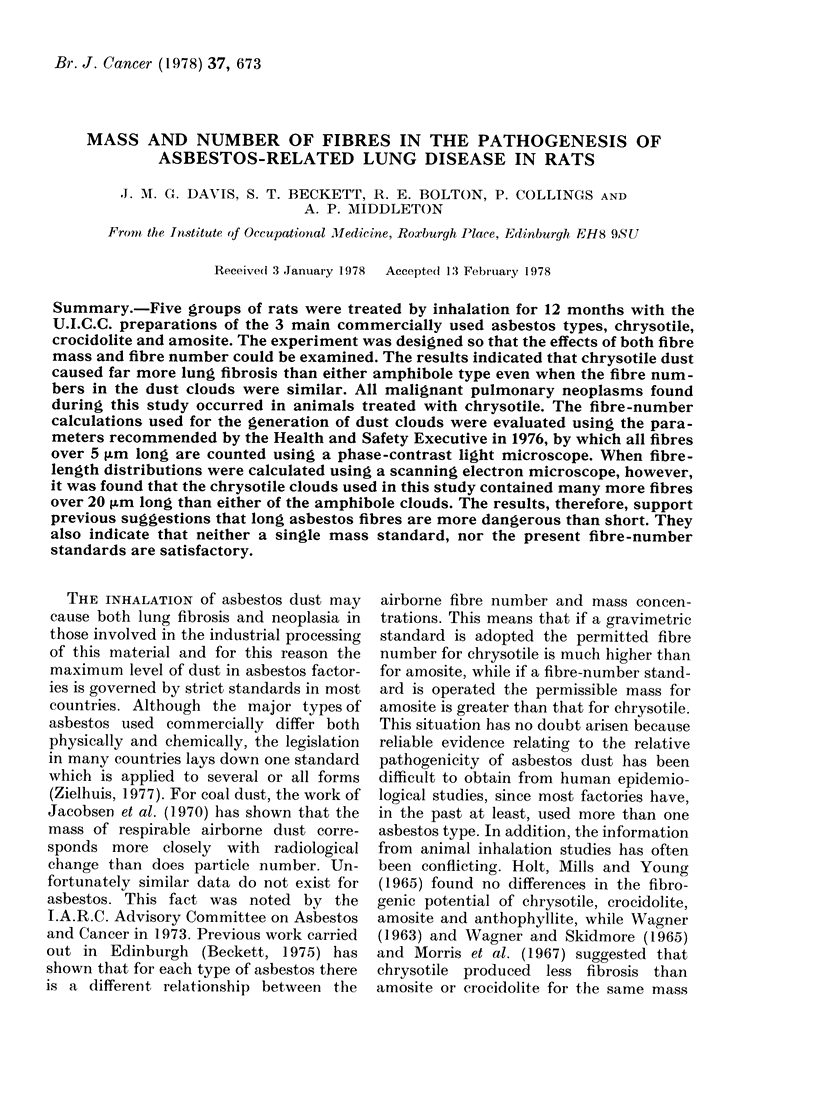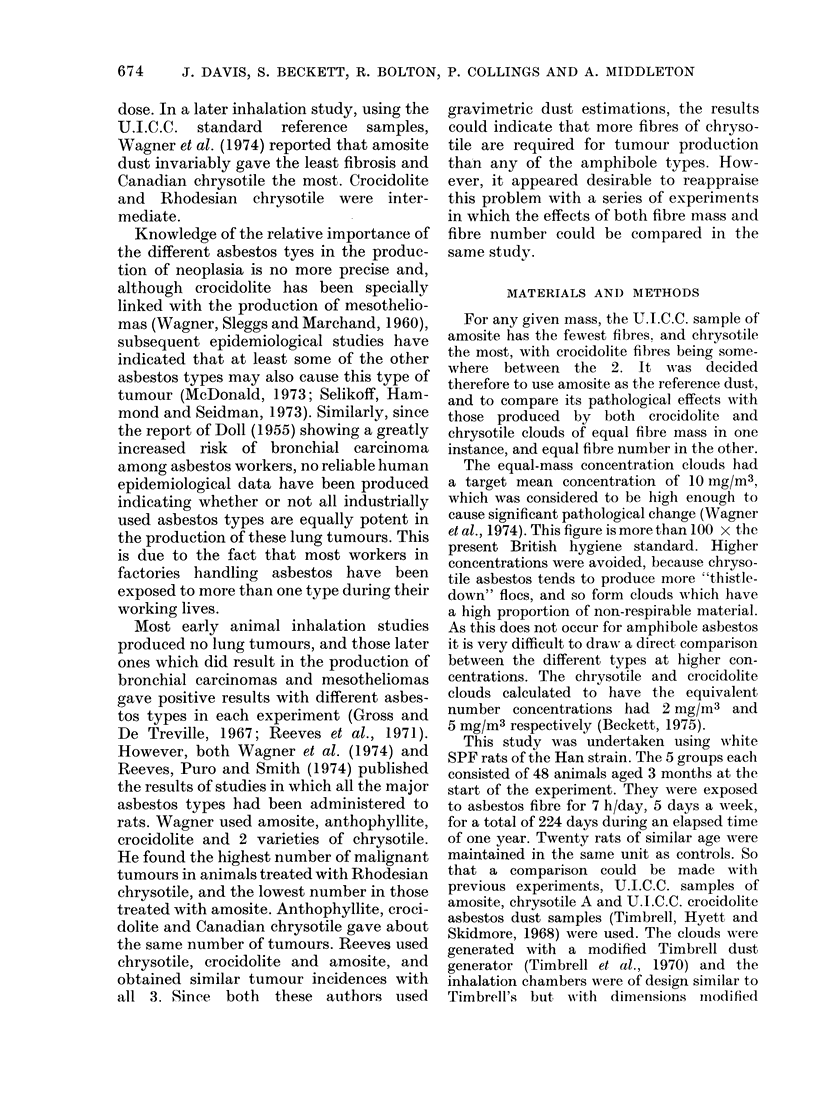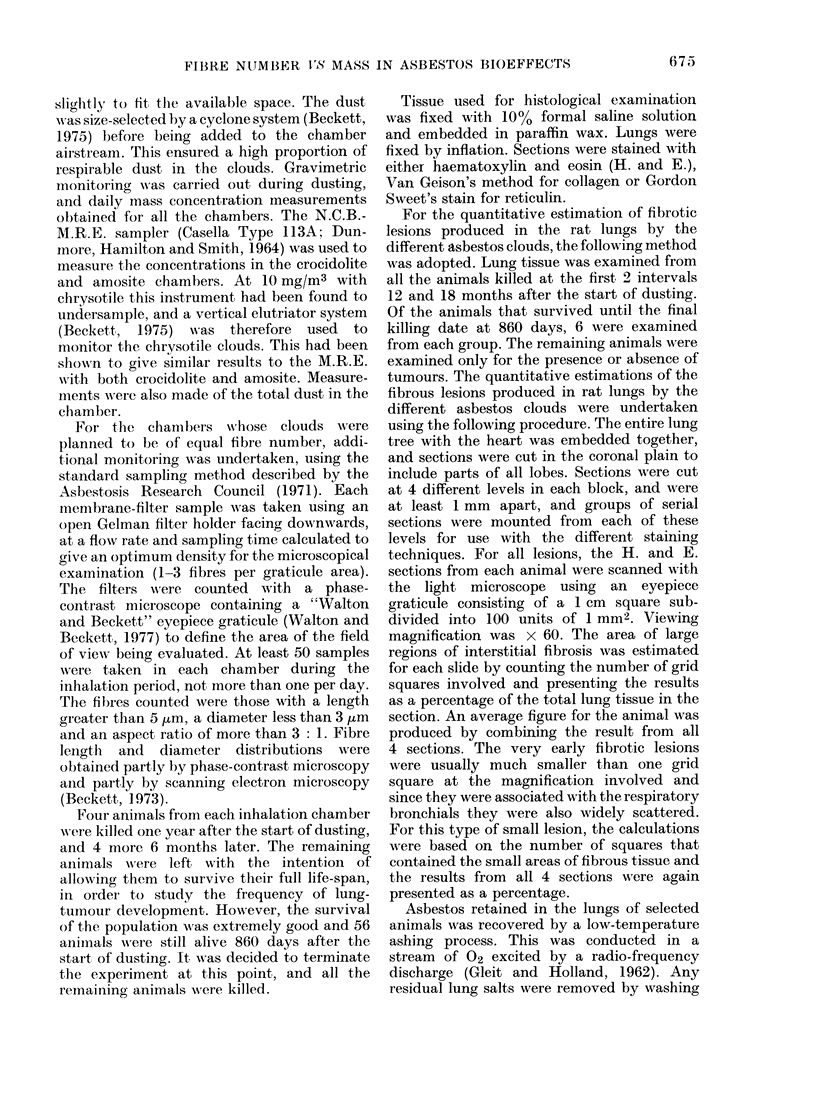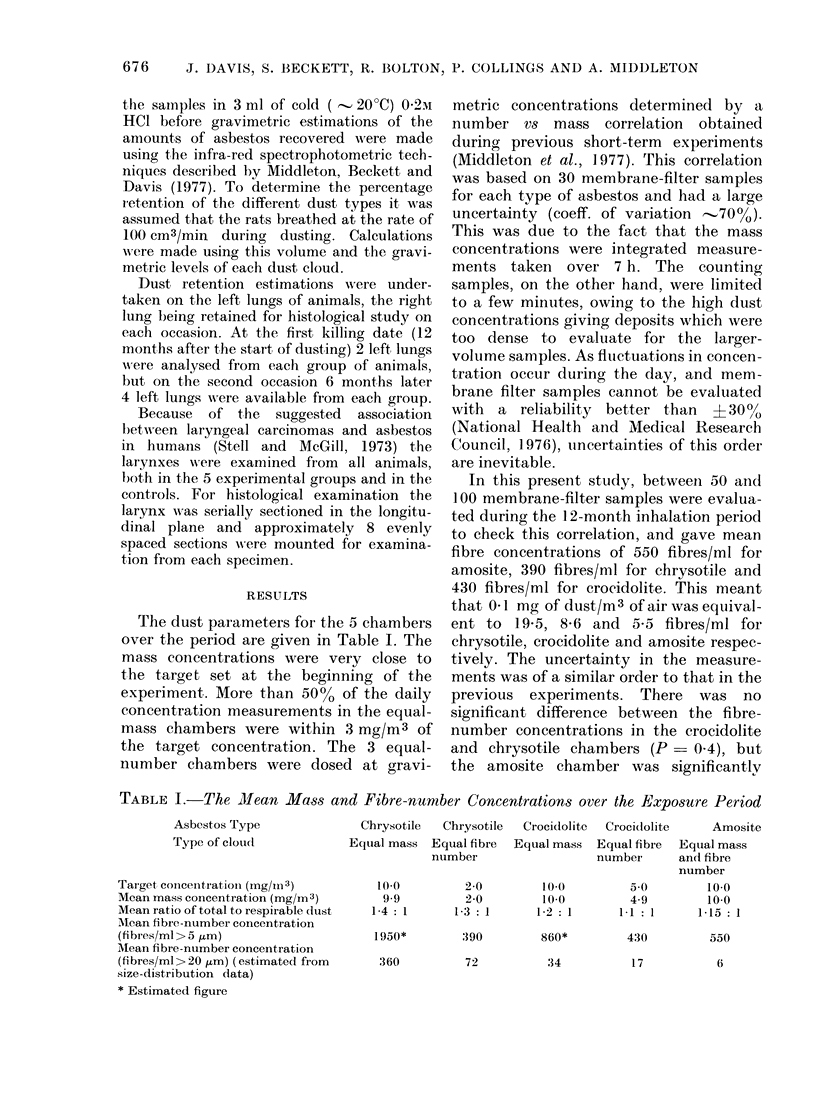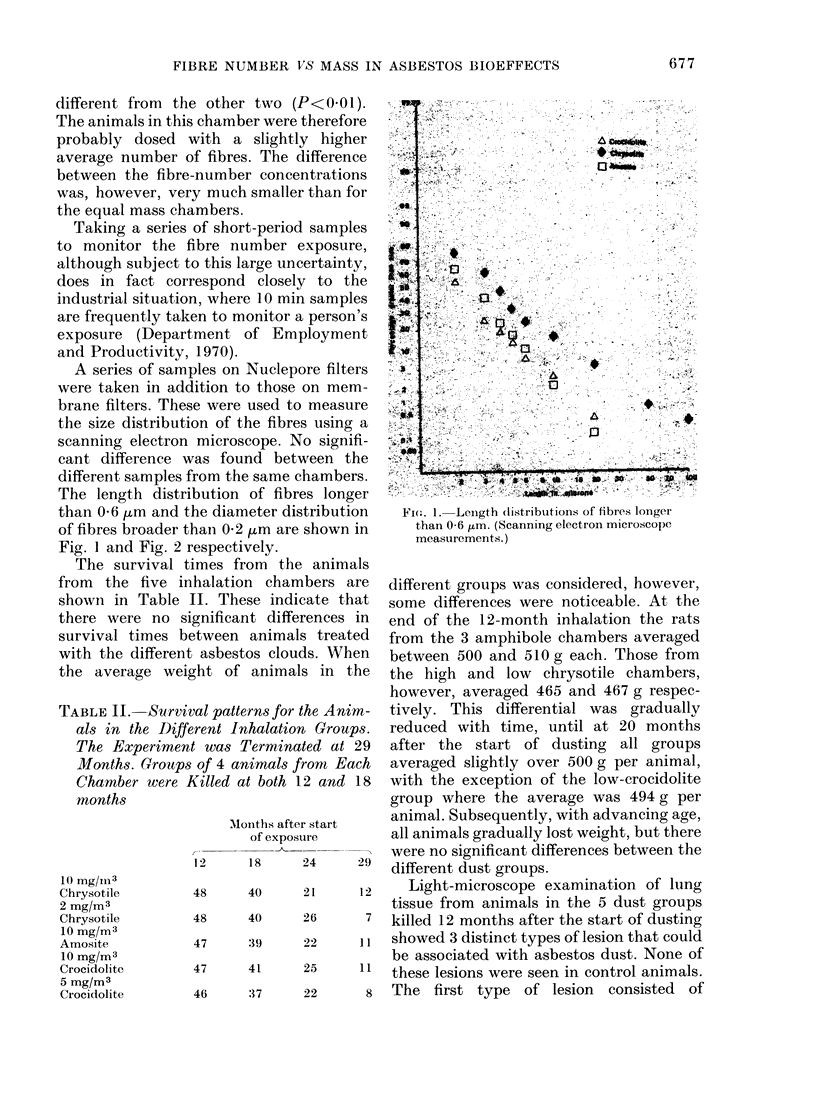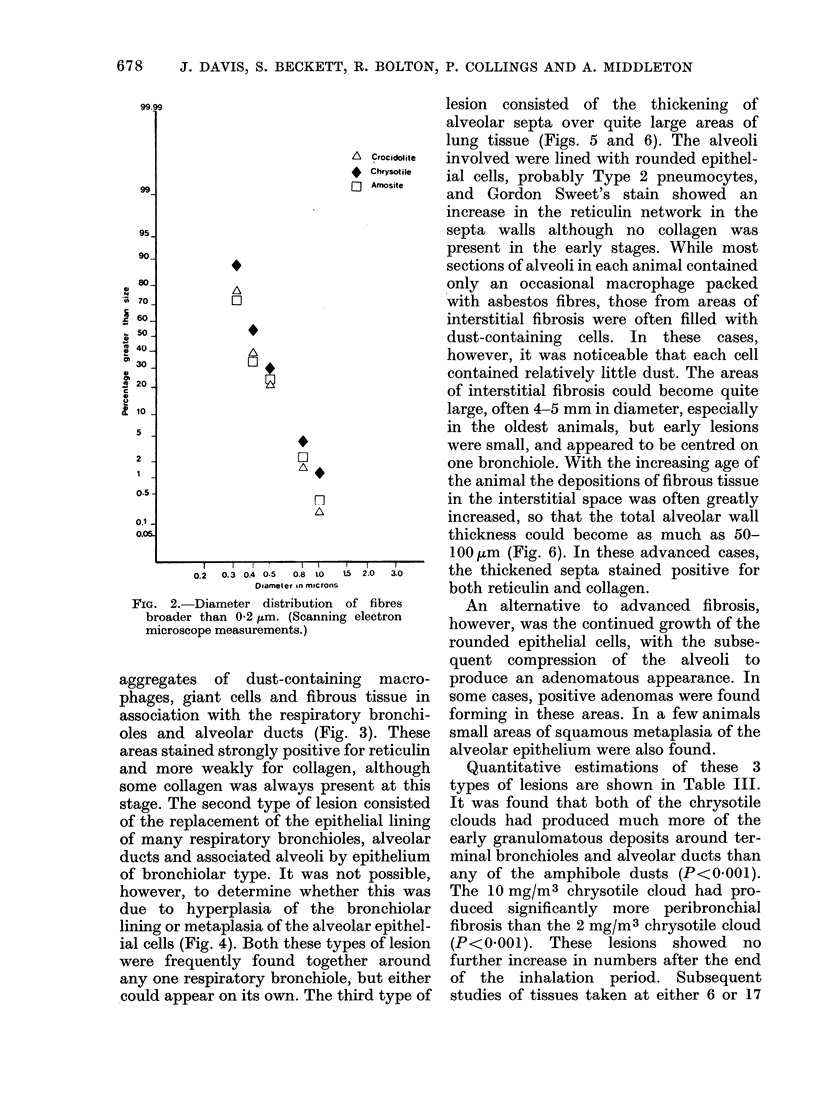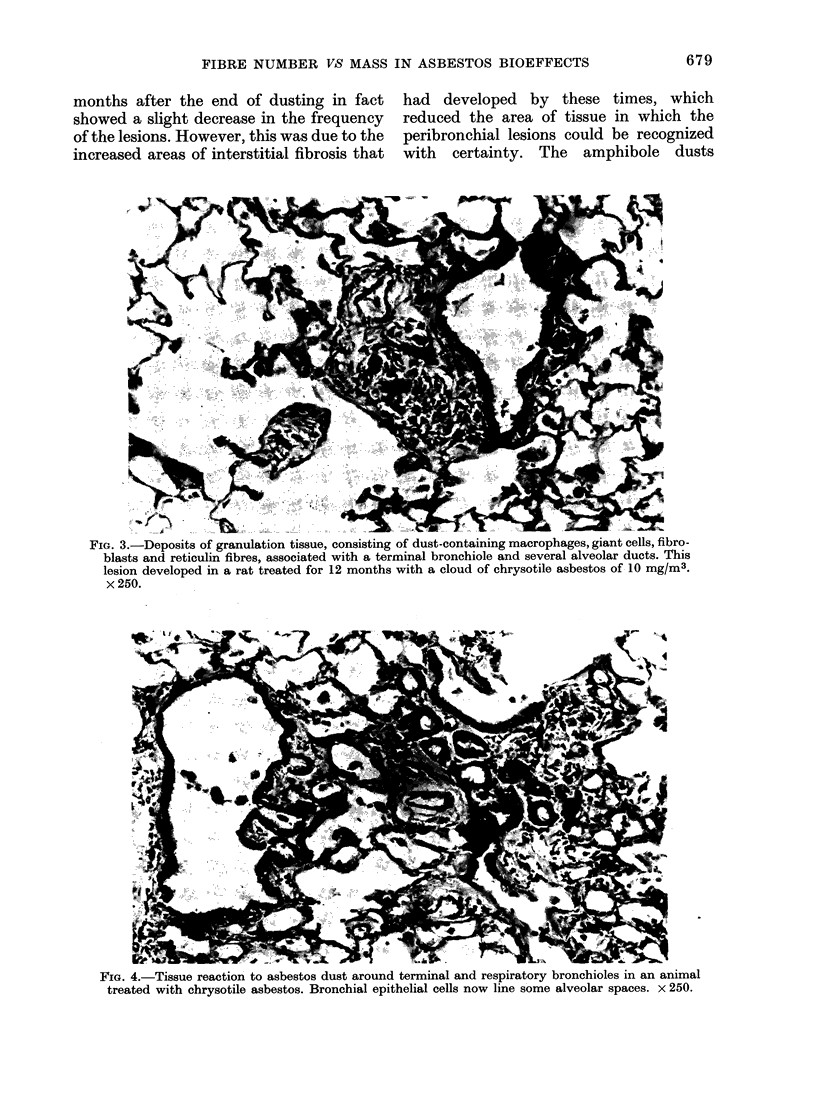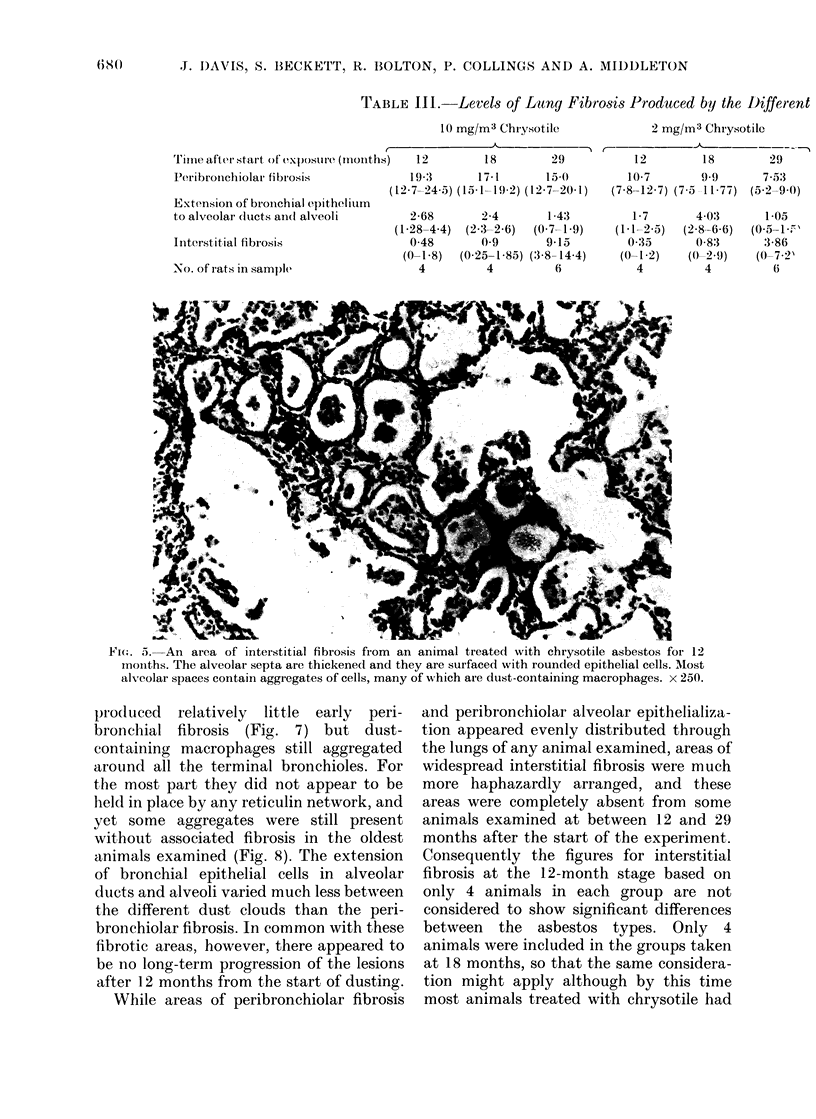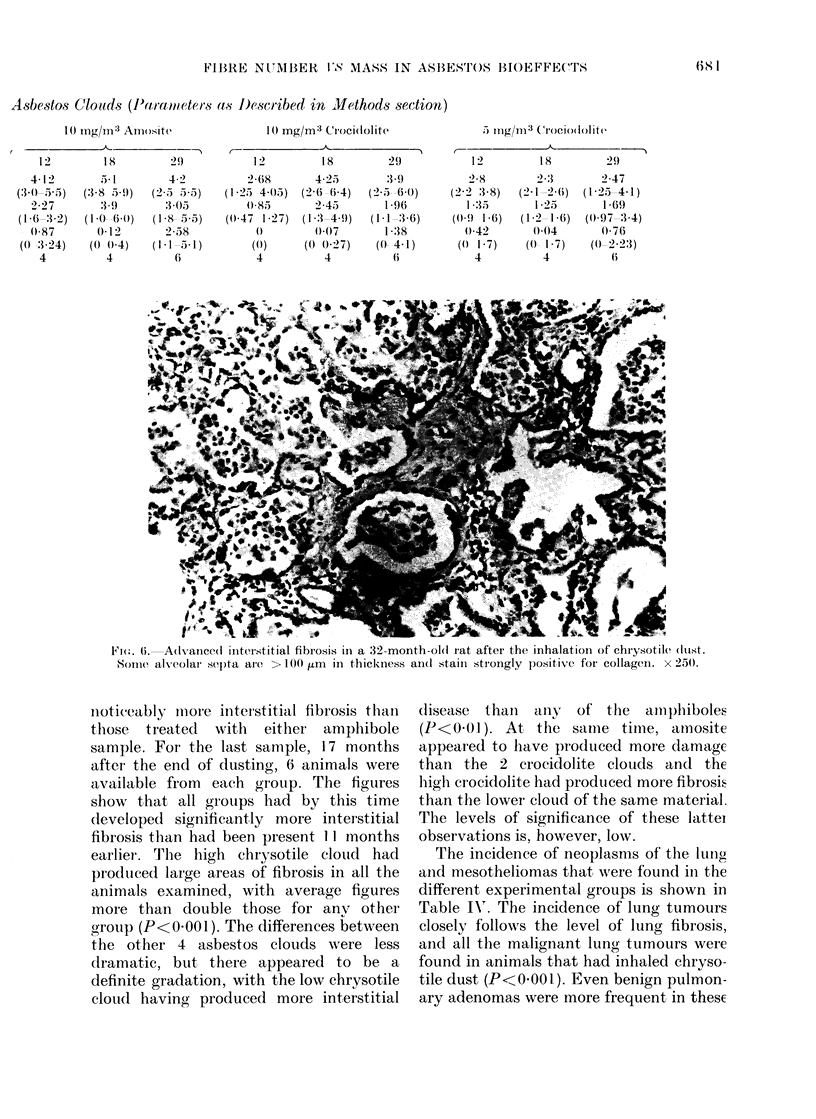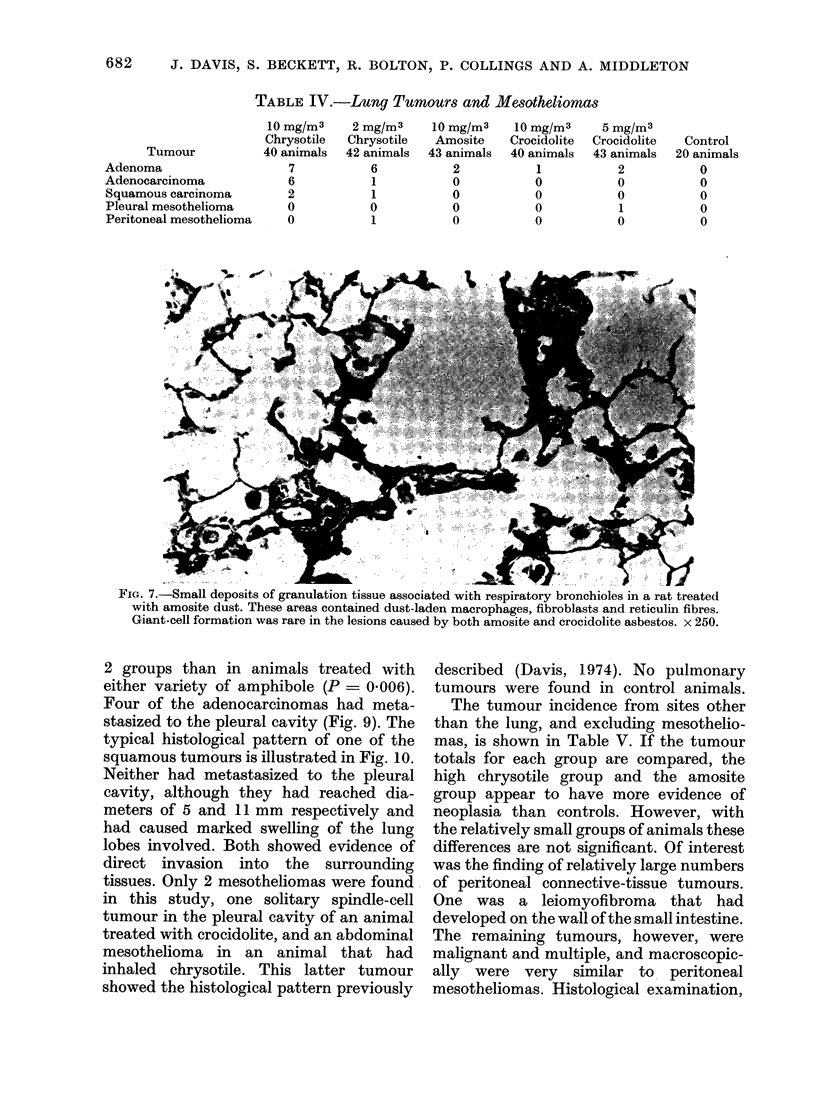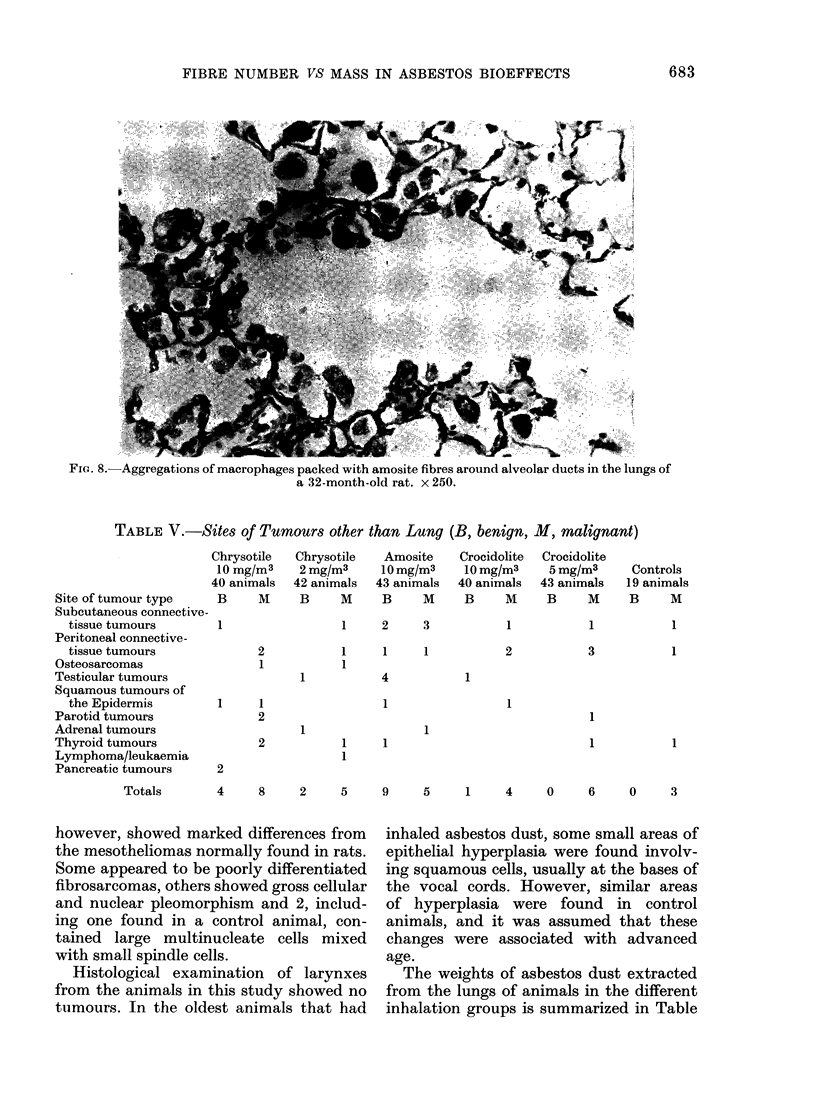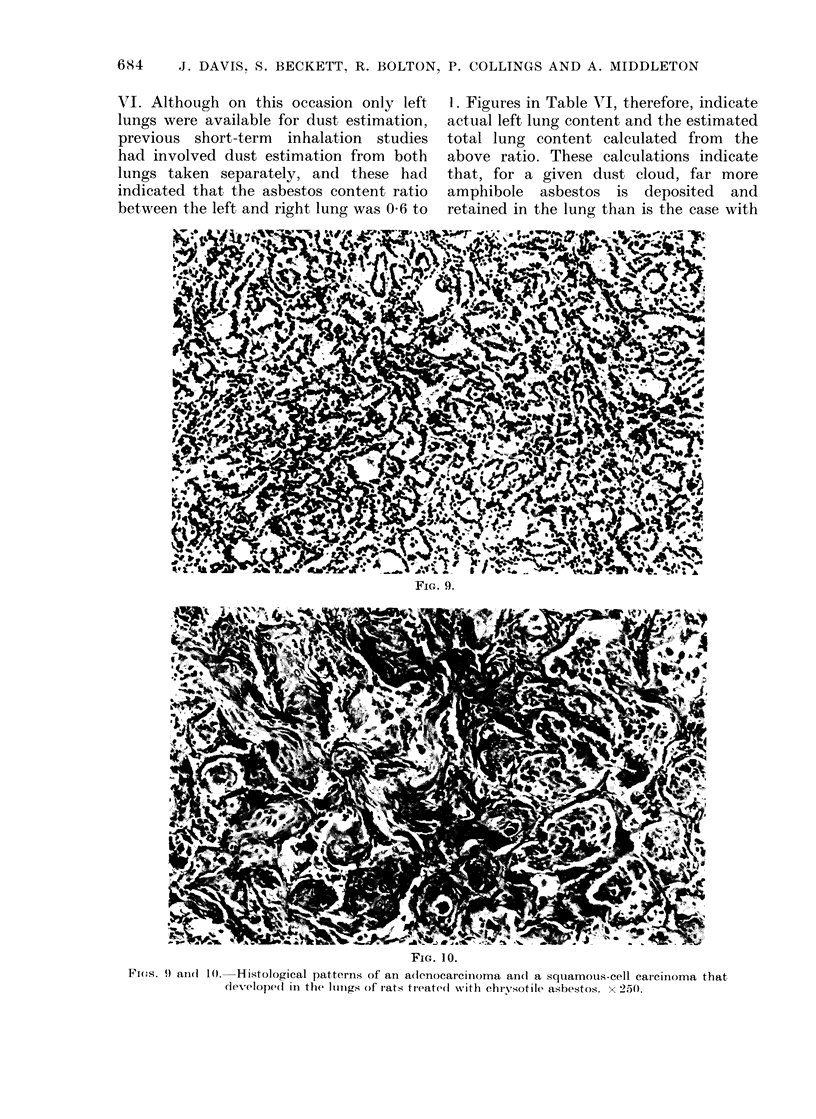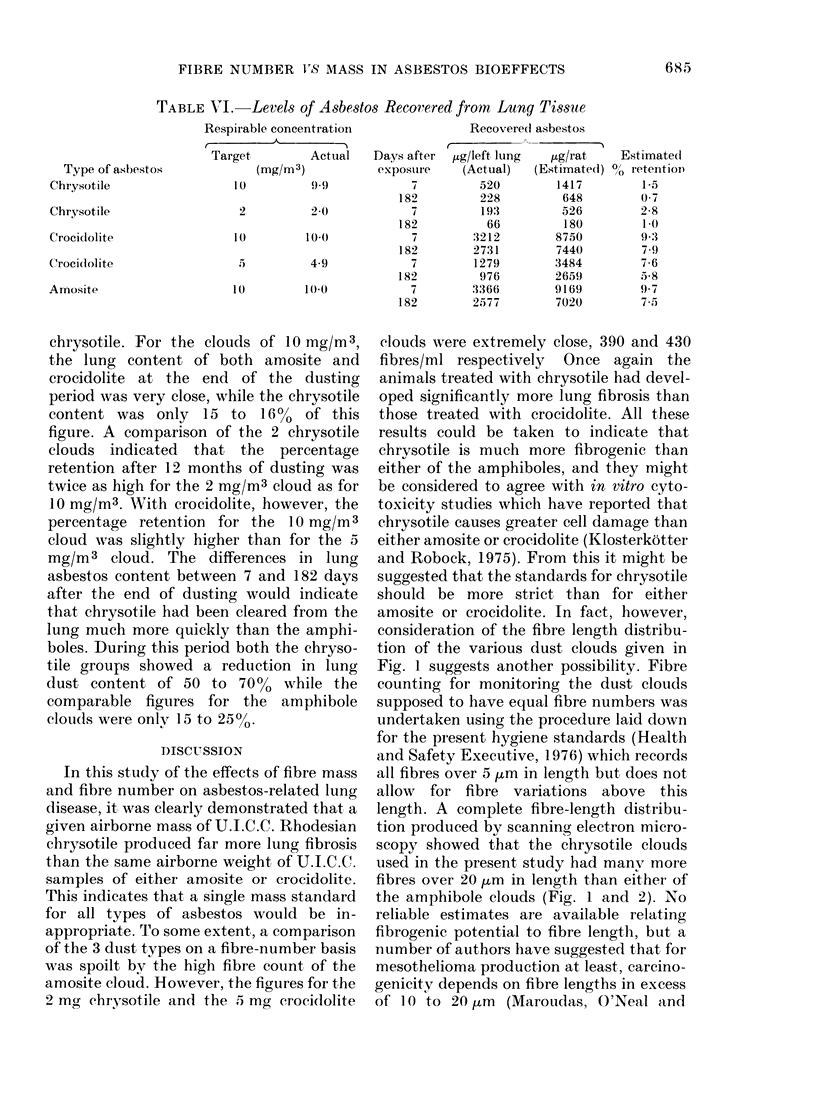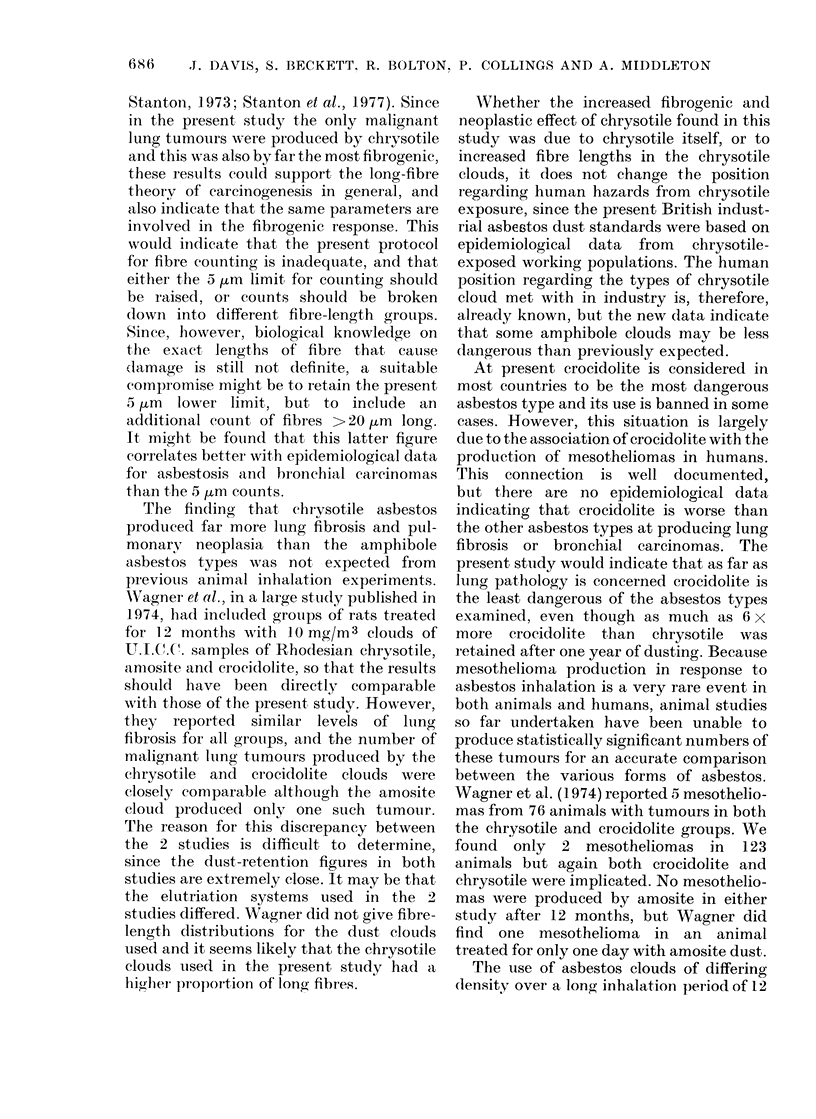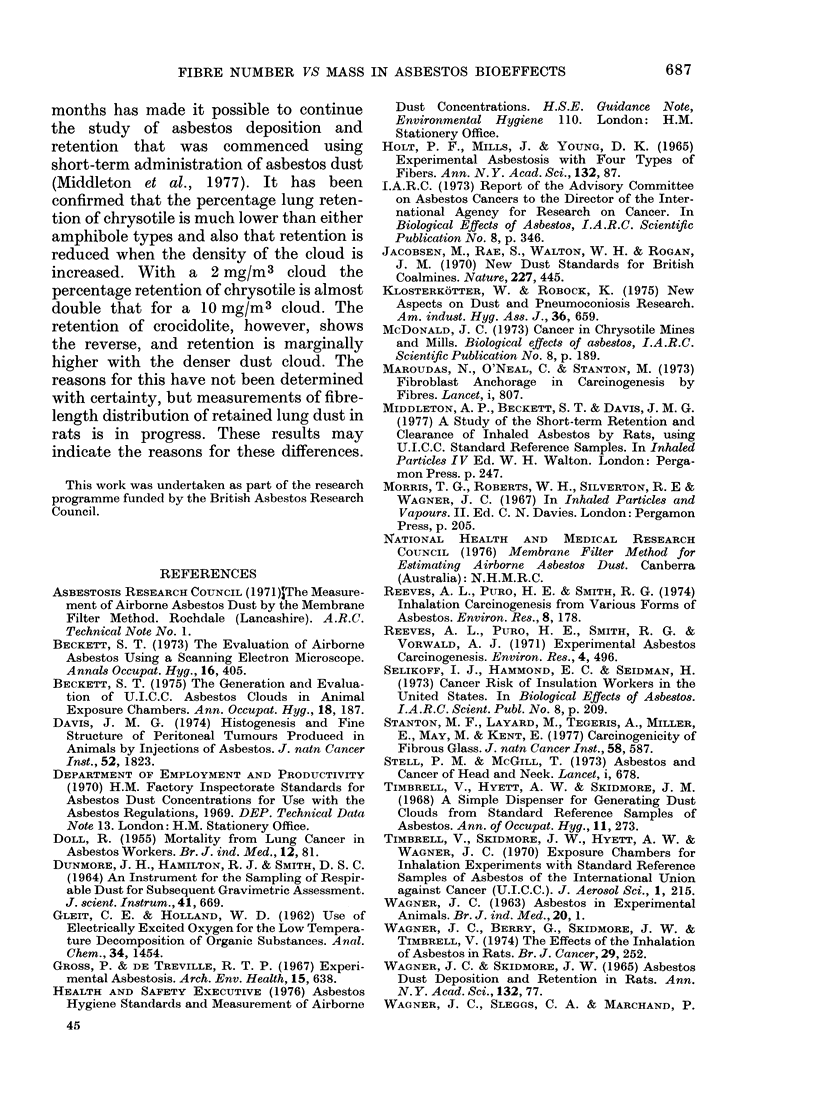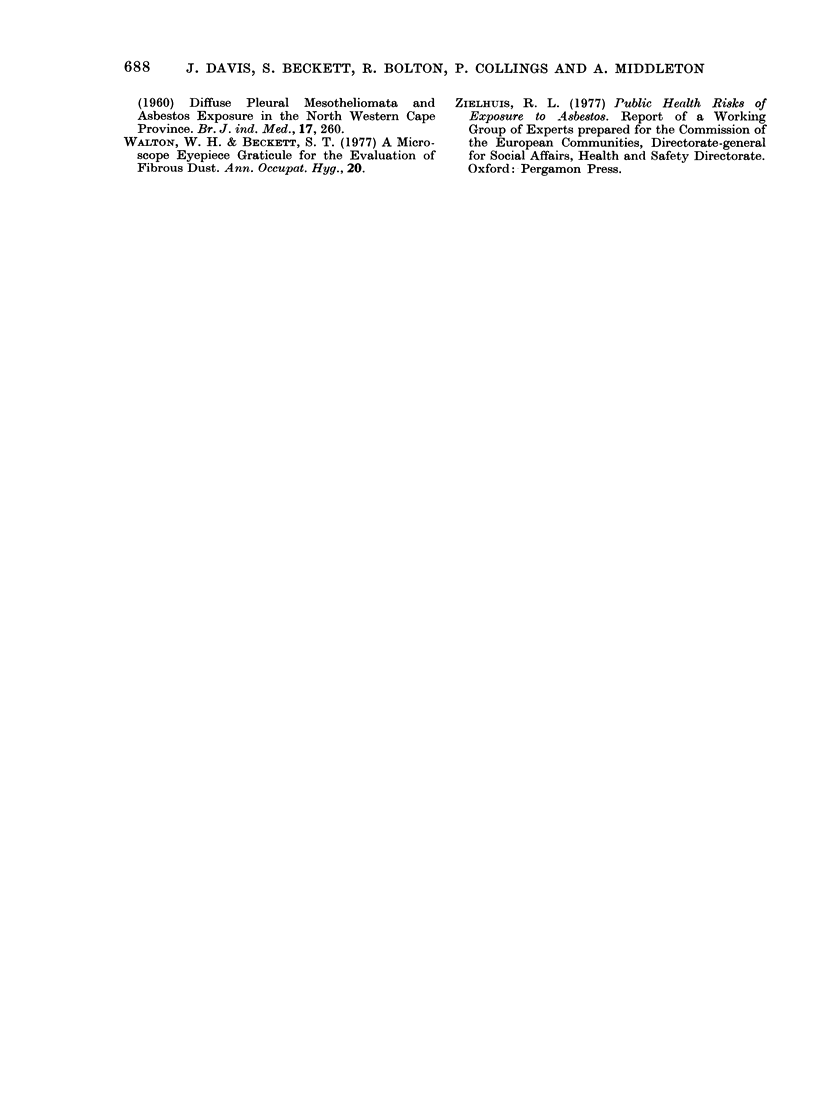# Mass and number of fibres in the pathogenesis of asbestos-related lung disease in rats.

**DOI:** 10.1038/bjc.1978.105

**Published:** 1978-05

**Authors:** J. M. Davis, S. T. Beckett, R. E. Bolton, P. Collings, A. P. Middleton

## Abstract

**Images:**


*Br. J. Cancer* (1978) **37**, 673

### MASS AND NUMBER OF FIBRES IN THE PATHOGENESIS OF ASBESTOS-RELATED LUNG DISEASE IN RATS

J. M. G. DAVIS, S. T. BECKETT, R. E. BOLTON, P. COLLINGS AND A. P. MIDDLETON

*From the Institute of Occupational Medicine, Roxburgh Place, Edinburgh EH8 9SU*

Received 3 January 1978 Accepted 13 February 1978

**Summary.—Five groups of rats were treated by inhalation for 12 months with the U.I.C.C. preparations of the 3 main commercially used asbestos types, chrysotile, crocidolite and amosite. The experiment was designed so that the effects of both fibre mass and fibre number could be examined. The results indicated that chrysotile dust caused far more lung fibrosis than either amphibole type even when the fibre numbers in the dust clouds were similar. All malignant pulmonary neoplasms found during this study occurred in animals treated with chrysotile. The fibre-number calculations used for the generation of dust clouds were evaluated using the parameters recommended by the Health and Safety Executive in 1976, by which all fibres over 5 μm long are counted using a phase-contrast light microscope. When fibre-length distributions were calculated using a scanning electron microscope, however, it was found that the chrysotile clouds used in this study contained many more fibres over 20 μm long than either of the amphibole clouds. The results, therefore, support previous suggestions that long asbestos fibres are more dangerous than short. They also indicate that neither a single mass standard, nor the present fibre-number standards are satisfactory.**

THE INHALATION of asbestos dust may cause both lung fibrosis and neoplasia in those involved in the industrial processing of this material and for this reason the maximum level of dust in asbestos factories is governed by strict standards in most countries. Although the major types of asbestos used commercially differ both physically and chemically, the legislation in many countries lays down one standard which is applied to several or all forms (Zielhuis, 1977). For coal dust, the work of Jacobsen *et al.* (1970) has shown that the mass of respirable airborne dust corresponds more closely with radiological change than does particle number. Unfortunately similar data do not exist for asbestos. This fact was noted by the I.A.R.C. Advisory Committee on Asbestos and Cancer in 1973. Previous work carried out in Edinburgh (Beckett, 1975) has shown that for each type of asbestos there is a different relationship between the airborne fibre number and mass concentrations. This means that if a gravimetric standard is adopted the permitted fibre number for chrysotile is much higher than for amosite, while if a fibre-number standard is operated the permissible mass for amosite is greater than that for chrysotile. This situation has no doubt arisen because reliable evidence relating to the relative pathogenicity of asbestos dust has been difficult to obtain from human epidemiological studies, since most factories have, in the past at least, used more than one asbestos type. In addition, the information from animal inhalation studies has often been conflicting. Holt, Mills and Young (1965) found no differences in the fibrogenic potential of chrysotile, crocidolite, amosite and anthophyllite, while Wagner (1963) and Wagner and Skidmore (1965) and Morris *et al.* (1967) suggested that chrysotile produced less fibrosis than amosite or crocidolite for the same mass dose. In a later inhalation study, using the U.I.C.C. standard reference samples, Wagner *et al.* (1974) reported that amosite dust invariably gave the least fibrosis and Canadian chrysotile the most. Crocidolite and Rhodesian chrysotile were intermediate.

Knowledge of the relative importance of the different asbestos tyes in the production of neoplasia is no more precise and, although crocidolite has been specially linked with the production of mesotheliomas (Wagner, Sleggs and Marchand, 1960), subsequent epidemiological studies have indicated that at least some of the other asbestos types may also cause this type of tumour (McDonald, 1973; Selikoff, Hammond and Seidman, 1973). Similarly, since the report of Doll (1955) showing a greatly increased risk of bronchial carcinoma among asbestos workers, no reliable human epidemiological data have been produced indicating whether or not all industrially used asbestos types are equally potent in the production of these lung tumours. This is due to the fact that most workers in factories handling asbestos have been exposed to more than one type during their working lives.

Most early animal inhalation studies produced no lung tumours, and those later ones which did result in the production of bronchial carcinomas and mesotheliomas gave positive results with different asbestos types in each experiment (Gross and De Treville, 1967; Reeves *et al.*, 1971). However, both Wagner *et al.* (1974) and Reeves, Puro and Smith (1974) published the results of studies in which all the major asbestos types had been administered to rats. Wagner used amosite, anthophyllite, crocidolite and 2 varieties of chrysotile. He found the highest number of malignant tumours in animals treated with Rhodesian chrysotile, and the lowest number in those treated with amosite. Anthophyllite, crocidolite and Canadian chrysotile gave about the same number of tumours. Reeves used chrysotile, crocidolite and amosite, and obtained similar tumour incidences with all 3. Since both these authors used gravimetric dust estimations, the results could indicate that more fibres of chrysotile are required for tumour production than any of the amphibole types. However, it appeared desirable to reappraise this problem with a series of experiments in which the effects of both fibre mass and fibre number could be compared in the same study.

### MATERIALS AND METHODS

For any given mass, the U.I.C.C. sample of amosite has the fewest fibres, and chrysotile the most, with crocidolite fibres being somewhere between the 2. It was decided therefore to use amosite as the reference dust, and to compare its pathological effects with those produced by both crocidolite and chrysotile clouds of equal fibre mass in one instance, and equal fibre number in the other.

The equal-mass concentration clouds had a target mean concentration of 10 mg/m$^3$, which was considered to be high enough to cause significant pathological change (Wagner *et al.*, 1974). This figure is more than 100 $\times$ the present British hygiene standard. Higher concentrations were avoided, because chrysotile asbestos tends to produce more "thistle-down" flocs, and so form clouds which have a high proportion of non-respirable material. As this does not occur for amphibole asbestos it is very difficult to draw a direct comparison between the different types at higher concentrations. The chrysotile and crocidolite clouds calculated to have the equivalent number concentrations had 2 mg/m$^3$ and 5 mg/m$^3$ respectively (Beckett, 1975).

This study was undertaken using white SPF rats of the Han strain. The 5 groups each consisted of 48 animals aged 3 months at the start of the experiment. They were exposed to asbestos fibre for 7 h/day, 5 days a week, for a total of 224 days during an elapsed time of one year. Twenty rats of similar age were maintained in the same unit as controls. So that a comparison could be made with previous experiments, U.I.C.C. samples of amosite, chrysotile A and U.I.C.C. crocidolite asbestos dust samples (Timbrell, Hyett and Skidmore, 1968) were used. The clouds were generated with a modified Timbrell dust generator (Timbrell *et al.*, 1970) and the inhalation chambers were of design similar to Timbrell's but with dimensions modified slightly to fit the available space. The dust was size-selected by a cyclone system (Beckett, 1975) before being added to the chamber airstream. This ensured a high proportion of respirable dust in the clouds. Gravimetric monitoring was carried out during dusting, and daily mass concentration measurements obtained for all the chambers. The N.C.B.-M.R.E. sampler (Casella Type 113A; Dunmore, Hamilton and Smith, 1964) was used to measure the concentrations in the crocidolite and amosite chambers. At 10 mg/m$^3$ with chrysotile this instrument had been found to undersample, and a vertical elutriator system (Beckett, 1975) was therefore used to monitor the chrysotile clouds. This had been shown to give similar results to the M.R.E. with both crocidolite and amosite. Measurements were also made of the total dust in the chamber.

For the chambers whose clouds were planned to be of equal fibre number, additional monitoring was undertaken, using the standard sampling method described by the Asbestosis Research Council (1971). Each membrane-filter sample was taken using an open Gelman filter holder facing downwards, at a flow rate and sampling time calculated to give an optimum density for the microscopical examination (1–3 fibres per graticule area). The filters were counted with a phase-contrast microscope containing a "Walton and Beckett" eyepiece graticule (Walton and Beckett, 1977) to define the area of the field of view being evaluated. At least 50 samples were taken in each chamber during the inhalation period, not more than one per day. The fibres counted were those with a length greater than 5 $\mu$m, a diameter less than 3 $\mu$m and an aspect ratio of more than 3 : 1. Fibre length and diameter distributions were obtained partly by phase-contrast microscopy and partly by scanning electron microscopy (Beckett, 1973).

Four animals from each inhalation chamber were killed one year after the start of dusting, and 4 more 6 months later. The remaining animals were left with the intention of allowing them to survive their full life-span, in order to study the frequency of lung-tumour development. However, the survival of the population was extremely good and 56 animals were still alive 860 days after the start of dusting. It was decided to terminate the experiment at this point, and all the remaining animals were killed.

Tissue used for histological examination was fixed with 10% formal saline solution and embedded in paraffin wax. Lungs were fixed by inflation. Sections were stained with either haematoxylin and eosin (H. and E.), Van Geison's method for collagen or Gordon Sweet's stain for reticulin.

For the quantitative estimation of fibrotic lesions produced in the rat lungs by the different asbestos clouds, the following method was adopted. Lung tissue was examined from all the animals killed at the first 2 intervals 12 and 18 months after the start of dusting. Of the animals that survived until the final killing date at 860 days, 6 were examined from each group. The remaining animals were examined only for the presence or absence of tumours. The quantitative estimations of the fibrous lesions produced in rat lungs by the different asbestos clouds were undertaken using the following procedure. The entire lung tree with the heart was embedded together, and sections were cut in the coronal plain to include parts of all lobes. Sections were cut at 4 different levels in each block, and were at least 1 mm apart, and groups of serial sections were mounted from each of these levels for use with the different staining techniques. For all lesions, the H. and E. sections from each animal were scanned with the light microscope using an eyepiece graticule consisting of a 1 cm square subdivided into 100 units of 1 mm$^2$. Viewing magnification was $\times$ 60. The area of large regions of interstitial fibrosis was estimated for each slide by counting the number of grid squares involved and presenting the results as a percentage of the total lung tissue in the section. An average figure for the animal was produced by combining the result from all 4 sections. The very early fibrotic lesions were usually much smaller than one grid square at the magnification involved and since they were associated with the respiratory bronchials they were also widely scattered. For this type of small lesion, the calculations were based on the number of squares that contained the small areas of fibrous tissue and the results from all 4 sections were again presented as a percentage.

Asbestos retained in the lungs of selected animals was recovered by a low-temperature ashing process. This was conducted in a stream of $O_2$ excited by a radio-frequency discharge (Gleit and Holland, 1962). Any residual lung salts were removed by washing the samples in 3 ml of cold ( ~20°C) 0·2M HCl before gravimetric estimations of the amounts of asbestos recovered were made using the infra-red spectrophotometric techniques described by Middleton, Beckett and Davis (1977). To determine the percentage retention of the different dust types it was assumed that the rats breathed at the rate of 100 cm$^3$/min during dusting. Calculations were made using this volume and the gravimetric levels of each dust cloud.

Dust retention estimations were undertaken on the left lungs of animals, the right lung being retained for histological study on each occasion. At the first killing date (12 months after the start of dusting) 2 left lungs were analysed from each group of animals, but on the second occasion 6 months later 4 left lungs were available from each group.

Because of the suggested association between laryngeal carcinomas and asbestos in humans (Stell and McGill, 1973) the larynxes were examined from all animals, both in the 5 experimental groups and in the controls. For histological examination the larynx was serially sectioned in the longitudinal plane and approximately 8 evenly spaced sections were mounted for examination from each specimen.

### RESULTS

The dust parameters for the 5 chambers over the period are given in Table I. The mass concentrations were very close to the target set at the beginning of the experiment. More than 50% of the daily concentration measurements in the equal-mass chambers were within 3 mg/m$^3$ of the target concentration. The 3 equal-number chambers were dosed at gravimetric concentrations determined by a number *vs* mass correlation obtained during previous short-term experiments (Middleton *et al.*, 1977). This correlation was based on 30 membrane-filter samples for each type of asbestos and had a large uncertainty (coeff. of variation ~70%). This was due to the fact that the mass concentrations were integrated measurements taken over 7 h. The counting samples, on the other hand, were limited to a few minutes, owing to the high dust concentrations giving deposits which were too dense to evaluate for the larger-volume samples. As fluctuations in concentration occur during the day, and membrane filter samples cannot be evaluated with a reliability better than ±30% (National Health and Medical Research Council, 1976), uncertainties of this order are inevitable.

In this present study, between 50 and 100 membrane-filter samples were evaluated during the 12-month inhalation period to check this correlation, and gave mean fibre concentrations of 550 fibres/ml for amosite, 390 fibres/ml for chrysotile and 430 fibres/ml for crocidolite. This meant that 0·1 mg of dust/m$^3$ of air was equivalent to 19·5, 8·6 and 5·5 fibres/ml for chrysotile, crocidolite and amosite respectively. The uncertainty in the measurements was of a similar order to that in the previous experiments. There was no significant difference between the fibre-number concentrations in the crocidolite and chrysotile chambers ($P = 0{\cdot}4$), but the amosite chamber was significantly

TABLE I.—*The Mean Mass and Fibre-number Concentrations over the Exposure Period*

| Asbestos Type | Chrysotile | Chrysotile | Crocidolite | Crocidolite | Amosite |
|---|---|---|---|---|---|
| Type of cloud | Equal mass | Equal fibre number | Equal mass | Equal fibre number | Equal mass and fibre number |
| Target concentration (mg/m$^3$) | 10·0 | 2·0 | 10·0 | 5·0 | 10·0 |
| Mean mass concentration (mg/m$^3$) | 9·9 | 2·0 | 10·0 | 4·9 | 10·0 |
| Mean ratio of total to respirable dust | 1·4 : 1 | 1·3 : 1 | 1·2 : 1 | 1·1 : 1 | 1·15 : 1 |
| Mean fibre-number concentration (fibres/ml > 5 μm) | 1950* | 390 | 860* | 430 | 550 |
| Mean fibre-number concentration (fibres/ml > 20 μm) (estimated from size-distribution data) | 360 | 72 | 34 | 17 | 6 |

* Estimated figure different from the other two ($P<0\cdot01$). The animals in this chamber were therefore probably dosed with a slightly higher average number of fibres. The difference between the fibre-number concentrations was, however, very much smaller than for the equal mass chambers.

Taking a series of short-period samples to monitor the fibre number exposure, although subject to this large uncertainty, does in fact correspond closely to the industrial situation, where 10 min samples are frequently taken to monitor a person's exposure (Department of Employment and Productivity, 1970).

A series of samples on Nuclepore filters were taken in addition to those on membrane filters. These were used to measure the size distribution of the fibres using a scanning electron microscope. No significant difference was found between the different samples from the same chambers. The length distribution of fibres longer than 0·6 μm and the diameter distribution of fibres broader than 0·2 μm are shown in Fig. 1 and Fig. 2 respectively.

The survival times from the animals from the five inhalation chambers are shown in Table II. These indicate that there were no significant differences in survival times between animals treated with the different asbestos clouds. When the average weight of animals in the

TABLE II.—*Survival patterns for the Animals in the Different Inhalation Groups. The Experiment was Terminated at* 29 *Months. Groups of* 4 *animals from Each Chamber were Killed at both* 12 *and* 18 *months*

| | Months after start of exposure | | | |
|---|---|---|---|---|
| | 12 | 18 | 24 | 29 |
| 10 mg/m³ Chrysotile | 48 | 40 | 21 | 12 |
| 2 mg/m³ Chrysotile | 48 | 40 | 26 | 7 |
| 10 mg/m³ Amosite | 47 | 39 | 22 | 11 |
| 10 mg/m³ Crocidolite | 47 | 41 | 25 | 11 |
| 5 mg/m³ Crocidolite | 46 | 37 | 22 | 8 |

FIG. 1.—Length distributions of fibres longer than 0·6 μm. (Scanning electron microscope measurements.)

different groups was considered, however, some differences were noticeable. At the end of the 12-month inhalation the rats from the 3 amphibole chambers averaged between 500 and 510 g each. Those from the high and low chrysotile chambers, however, averaged 465 and 467 g respectively. This differential was gradually reduced with time, until at 20 months after the start of dusting all groups averaged slightly over 500 g per animal, with the exception of the low-crocidolite group where the average was 494 g per animal. Subsequently, with advancing age, all animals gradually lost weight, but there were no significant differences between the different dust groups.

Light-microscope examination of lung tissue from animals in the 5 dust groups killed 12 months after the start of dusting showed 3 distinct types of lesion that could be associated with asbestos dust. None of these lesions were seen in control animals. The first type of lesion consisted of

FIG. 2.—Diameter distribution of fibres broader than 0·2 $\mu$m. (Scanning electron microscope measurements.)

aggregates of dust-containing macrophages, giant cells and fibrous tissue in association with the respiratory bronchioles and alveolar ducts (Fig. 3). These areas stained strongly positive for reticulin and more weakly for collagen, although some collagen was always present at this stage. The second type of lesion consisted of the replacement of the epithelial lining of many respiratory bronchioles, alveolar ducts and associated alveoli by epithelium of bronchiolar type. It was not possible, however, to determine whether this was due to hyperplasia of the bronchiolar lining or metaplasia of the alveolar epithelial cells (Fig. 4). Both these types of lesion were frequently found together around any one respiratory bronchiole, but either could appear on its own. The third type of lesion consisted of the thickening of alveolar septa over quite large areas of lung tissue (Figs. 5 and 6). The alveoli involved were lined with rounded epithelial cells, probably Type 2 pneumocytes, and Gordon Sweet's stain showed an increase in the reticulin network in the septa walls although no collagen was present in the early stages. While most sections of alveoli in each animal contained only an occasional macrophage packed with asbestos fibres, those from areas of interstitial fibrosis were often filled with dust-containing cells. In these cases, however, it was noticeable that each cell contained relatively little dust. The areas of interstitial fibrosis could become quite large, often 4–5 mm in diameter, especially in the oldest animals, but early lesions were small, and appeared to be centred on one bronchiole. With the increasing age of the animal the depositions of fibrous tissue in the interstitial space was often greatly increased, so that the total alveolar wall thickness could become as much as 50–100 $\mu$m (Fig. 6). In these advanced cases, the thickened septa stained positive for both reticulin and collagen.

An alternative to advanced fibrosis, however, was the continued growth of the rounded epithelial cells, with the subsequent compression of the alveoli to produce an adenomatous appearance. In some cases, positive adenomas were found forming in these areas. In a few animals small areas of squamous metaplasia of the alveolar epithelium were also found.

Quantitative estimations of these 3 types of lesions are shown in Table III. It was found that both of the chrysotile clouds had produced much more of the early granulomatous deposits around terminal bronchioles and alveolar ducts than any of the amphibole dusts ($P<0·001$). The 10 mg/m$^3$ chrysotile cloud had produced significantly more peribronchial fibrosis than the 2 mg/m$^3$ chrysotile cloud ($P<0·001$). These lesions showed no further increase in numbers after the end of the inhalation period. Subsequent studies of tissues taken at either 6 or 17

months after the end of dusting in fact showed a slight decrease in the frequency of the lesions. However, this was due to the increased areas of interstitial fibrosis that had developed by these times, which reduced the area of tissue in which the peribronchial lesions could be recognized with certainty. The amphibole dusts

FIG. 3.—Deposits of granulation tissue, consisting of dust-containing macrophages, giant cells, fibroblasts and reticulin fibres, associated with a terminal bronchiole and several alveolar ducts. This lesion developed in a rat treated for 12 months with a cloud of chrysotile asbestos of 10 mg/m$^3$. $\times$250.

FIG. 4.—Tissue reaction to asbestos dust around terminal and respiratory bronchioles in an animal treated with chrysotile asbestos. Bronchial epithelial cells now line some alveolar spaces. $\times$250.

TABLE III.—*Levels of Lung Fibrosis Produced by the Different*

| | 10 mg/m³ Chrysotile | | | 2 mg/m³ Chrysotile | | |
|---|---|---|---|---|---|---|
| Time after start of exposure (months) | 12 | 18 | 29 | 12 | 18 | 29 |
| Peribronchiolar fibrosis | 19·3 (12·7–24·5) | 17·1 (15·1–19·2) | 15·0 (12·7–20·1) | 10·7 (7·8–12·7) | 9·9 (7·5–11·77) | 7·53 (5·2–9·0) |
| Extension of bronchial epithelium to alveolar ducts and alveoli | 2·68 (1·28–4·4) | 2·4 (2·3–2·6) | 1·43 (0·7–1·9) | 1·7 (1·1–2·5) | 4·03 (2·8–6·6) | 1·05 (0·5–1·5) |
| Interstitial fibrosis | 0·48 (0–1·8) | 0·9 (0·25–1·85) | 9·15 (3·8–14·4) | 0·35 (0–1·2) | 0·83 (0–2·9) | 3·86 (0–7·2) |
| No. of rats in sample | 4 | 4 | 6 | 4 | 4 | 6 |

FIG. 5.—An area of interstitial fibrosis from an animal treated with chrysotile asbestos for 12 months. The alveolar septa are thickened and they are surfaced with rounded epithelial cells. Most alveolar spaces contain aggregates of cells, many of which are dust-containing macrophages. ×250.

produced relatively little early peribronchial fibrosis (Fig. 7) but dust-containing macrophages still aggregated around all the terminal bronchioles. For the most part they did not appear to be held in place by any reticulin network, and yet some aggregates were still present without associated fibrosis in the oldest animals examined (Fig. 8). The extension of bronchial epithelial cells in alveolar ducts and alveoli varied much less between the different dust clouds than the peribronchiolar fibrosis. In common with these fibrotic areas, however, there appeared to be no long-term progression of the lesions after 12 months from the start of dusting.

While areas of peribronchiolar fibrosis and peribronchiolar alveolar epithelialization appeared evenly distributed through the lungs of any animal examined, areas of widespread interstitial fibrosis were much more haphazardly arranged, and these areas were completely absent from some animals examined at between 12 and 29 months after the start of the experiment. Consequently the figures for interstitial fibrosis at the 12-month stage based on only 4 animals in each group are not considered to show significant differences between the asbestos types. Only 4 animals were included in the groups taken at 18 months, so that the same consideration might apply although by this time most animals treated with chrysotile had

*Asbestos Clouds (Parameters as Described in Methods section)*

| 10 mg/m³ Amosite | | | 10 mg/m³ Crocidolite | | | 5 mg/m³ Crociodolite | | |
|---|---|---|---|---|---|---|---|---|
| 12 | 18 | 29 | 12 | 18 | 29 | 12 | 18 | 29 |
| 4·12 | 5·1 | 4·2 | 2·68 | 4·25 | 3·9 | 2·8 | 2·3 | 2·47 |
| (3·0–5·5) | (3·8–5·9) | (2·5–5·5) | (1·25–4·05) | (2·6–6·4) | (2·5–6·0) | (2·2–3·8) | (2·1–2·6) | (1·25–4·1) |
| 2·27 | 3·9 | 3·05 | 0·85 | 2·45 | 1·96 | 1·35 | 1·25 | 1·69 |
| (1·6–3·2) | (1·0–6·0) | (1·8–5·5) | (0·47–1·27) | (1·3–4·9) | (1·1–3·6) | (0·9–1·6) | (1·2–1·6) | (0·97–3·4) |
| 0·87 | 0·12 | 2·58 | 0 | 0·07 | 1·38 | 0·42 | 0·04 | 0·76 |
| (0–3·24) | (0–0·4) | (1·1–5·1) | (0) | (0–0·27) | (0–4·1) | (0–1·7) | (0–1·7) | (0–2·23) |
| 4 | 4 | 6 | 4 | 4 | 6 | 4 | 4 | 6 |

FIG. 6.—Advanced interstitial fibrosis in a 32-month-old rat after the inhalation of chrysotile dust. Some alveolar septa are >100 μm in thickness and stain strongly positive for collagen. ×250.

noticeably more interstitial fibrosis than those treated with either amphibole sample. For the last sample, 17 months after the end of dusting, 6 animals were available from each group. The figures show that all groups had by this time developed significantly more interstitial fibrosis than had been present 11 months earlier. The high chrysotile cloud had produced large areas of fibrosis in all the animals examined, with average figures more than double those for any other group ($P<0·001$). The differences between the other 4 asbestos clouds were less dramatic, but there appeared to be a definite gradation, with the low chrysotile cloud having produced more interstitial disease than any of the amphiboles ($P<0·01$). At the same time, amosite appeared to have produced more damage than the 2 crocidolite clouds and the high crocidolite had produced more fibrosis than the lower cloud of the same material. The levels of significance of these latter observations is, however, low.

The incidence of neoplasms of the lung and mesotheliomas that were found in the different experimental groups is shown in Table IV. The incidence of lung tumours closely follows the level of lung fibrosis, and all the malignant lung tumours were found in animals that had inhaled chrysotile dust ($P<0·001$). Even benign pulmonary adenomas were more frequent in these

TABLE IV.—*Lung Tumours and Mesotheliomas*

| Tumour | 10 mg/m³ Chrysotile 40 animals | 2 mg/m³ Chrysotile 42 animals | 10 mg/m³ Amosite 43 animals | 10 mg/m³ Crocidolite 40 animals | 5 mg/m³ Crocidolite 43 animals | Control 20 animals |
|---|---|---|---|---|---|---|
| Adenoma | 7 | 6 | 2 | 1 | 2 | 0 |
| Adenocarcinoma | 6 | 1 | 0 | 0 | 0 | 0 |
| Squamous carcinoma | 2 | 1 | 0 | 0 | 0 | 0 |
| Pleural mesothelioma | 0 | 0 | 0 | 0 | 1 | 0 |
| Peritoneal mesothelioma | 0 | 1 | 0 | 0 | 0 | 0 |

FIG. 7.—Small deposits of granulation tissue associated with respiratory bronchioles in a rat treated with amosite dust. These areas contained dust-laden macrophages, fibroblasts and reticulin fibres. Giant-cell formation was rare in the lesions caused by both amosite and crocidolite asbestos. × 250.

2 groups than in animals treated with either variety of amphibole ($P = 0{\cdot}006$). Four of the adenocarcinomas had metastasized to the pleural cavity (Fig. 9). The typical histological pattern of one of the squamous tumours is illustrated in Fig. 10. Neither had metastasized to the pleural cavity, although they had reached diameters of 5 and 11 mm respectively and had caused marked swelling of the lung lobes involved. Both showed evidence of direct invasion into the surrounding tissues. Only 2 mesotheliomas were found in this study, one solitary spindle-cell tumour in the pleural cavity of an animal treated with crocidolite, and an abdominal mesothelioma in an animal that had inhaled chrysotile. This latter tumour showed the histological pattern previously described (Davis, 1974). No pulmonary tumours were found in control animals.

The tumour incidence from sites other than the lung, and excluding mesotheliomas, is shown in Table V. If the tumour totals for each group are compared, the high chrysotile group and the amosite group appear to have more evidence of neoplasia than controls. However, with the relatively small groups of animals these differences are not significant. Of interest was the finding of relatively large numbers of peritoneal connective-tissue tumours. One was a leiomyofibroma that had developed on the wall of the small intestine. The remaining tumours, however, were malignant and multiple, and macroscopically were very similar to peritoneal mesotheliomas. Histological examination,

FIG. 8.—Aggregations of macrophages packed with amosite fibres around alveolar ducts in the lungs of a 32-month-old rat. ×250.

TABLE V.—*Sites of Tumours other than Lung* (*B, benign, M, malignant*)

| | Chrysotile 10 mg/m³ 40 animals | | Chrysotile 2 mg/m³ 42 animals | | Amosite 10 mg/m³ 43 animals | | Crocidolite 10 mg/m³ 40 animals | | Crocidolite 5 mg/m³ 43 animals | | Controls 19 animals | |
|---|---|---|---|---|---|---|---|---|---|---|---|---|
| Site of tumour type | B | M | B | M | B | M | B | M | B | M | B | M |
| Subcutaneous connective-tissue tumours | 1 | | | 1 | 2 | 3 | | 1 | | 1 | | 1 |
| Peritoneal connective-tissue tumours | | 2 | | 1 | 1 | 1 | | 2 | | 3 | | 1 |
| Osteosarcomas | | 1 | | 1 | | | | | | | | |
| Testicular tumours | | | 1 | | 4 | | 1 | | | | | |
| Squamous tumours of the Epidermis | 1 | 1 | | | 1 | | | 1 | | | | |
| Parotid tumours | | 2 | | | | | | | | 1 | | |
| Adrenal tumours | | | 1 | | | 1 | | | | | | |
| Thyroid tumours | | 2 | | 1 | 1 | | | | | 1 | | 1 |
| Lymphoma/leukaemia | | | | 1 | | | | | | | | |
| Pancreatic tumours | 2 | | | | | | | | | | | |
| Totals | 4 | 8 | 2 | 5 | 9 | 5 | 1 | 4 | 0 | 6 | 0 | 3 |

however, showed marked differences from the mesotheliomas normally found in rats. Some appeared to be poorly differentiated fibrosarcomas, others showed gross cellular and nuclear pleomorphism and 2, including one found in a control animal, contained large multinucleate cells mixed with small spindle cells.

Histological examination of larynxes from the animals in this study showed no tumours. In the oldest animals that had inhaled asbestos dust, some small areas of epithelial hyperplasia were found involving squamous cells, usually at the bases of the vocal cords. However, similar areas of hyperplasia were found in control animals, and it was assumed that these changes were associated with advanced age.

The weights of asbestos dust extracted from the lungs of animals in the different inhalation groups is summarized in Table

VI. Although on this occasion only left lungs were available for dust estimation, previous short-term inhalation studies had involved dust estimation from both lungs taken separately, and these had indicated that the asbestos content ratio between the left and right lung was 0·6 to 1. Figures in Table VI, therefore, indicate actual left lung content and the estimated total lung content calculated from the above ratio. These calculations indicate that, for a given dust cloud, far more amphibole asbestos is deposited and retained in the lung than is the case with

FIG. 9.

FIG. 10.

FIGS. 9 and 10.—Histological patterns of an adenocarcinoma and a squamous-cell carcinoma that developed in the lungs of rats treated with chrysotile asbestos. ×250.

TABLE VI.—*Levels of Asbestos Recovered from Lung Tissue*

| Type of asbestos | Respirable concentration (mg/m³) Target | Actual | Days after exposure | Recovered asbestos µg/left lung (Actual) | µg/rat (Estimated) | Estimated % retention |
|---|---|---|---|---|---|---|
| Chrysotile | 10 | 9·9 | 7 | 520 | 1417 | 1·5 |
| | | | 182 | 228 | 648 | 0·7 |
| Chrysotile | 2 | 2·0 | 7 | 193 | 526 | 2·8 |
| | | | 182 | 66 | 180 | 1·0 |
| Crocidolite | 10 | 10·0 | 7 | 3212 | 8750 | 9·3 |
| | | | 182 | 2731 | 7440 | 7·9 |
| Crocidolite | 5 | 4·9 | 7 | 1279 | 3484 | 7·6 |
| | | | 182 | 976 | 2659 | 5·8 |
| Amosite | 10 | 10·0 | 7 | 3366 | 9169 | 9·7 |
| | | | 182 | 2577 | 7020 | 7·5 |

chrysotile. For the clouds of 10 mg/m³, the lung content of both amosite and crocidolite at the end of the dusting period was very close, while the chrysotile content was only 15 to 16% of this figure. A comparison of the 2 chrysotile clouds indicated that the percentage retention after 12 months of dusting was twice as high for the 2 mg/m³ cloud as for 10 mg/m³. With crocidolite, however, the percentage retention for the 10 mg/m³ cloud was slightly higher than for the 5 mg/m³ cloud. The differences in lung asbestos content between 7 and 182 days after the end of dusting would indicate that chrysotile had been cleared from the lung much more quickly than the amphiboles. During this period both the chrysotile groups showed a reduction in lung dust content of 50 to 70% while the comparable figures for the amphibole clouds were only 15 to 25%.

### DISCUSSION

In this study of the effects of fibre mass and fibre number on asbestos-related lung disease, it was clearly demonstrated that a given airborne mass of U.I.C.C. Rhodesian chrysotile produced far more lung fibrosis than the same airborne weight of U.I.C.C. samples of either amosite or crocidolite. This indicates that a single mass standard for all types of asbestos would be inappropriate. To some extent, a comparison of the 3 dust types on a fibre-number basis was spoilt by the high fibre count of the amosite cloud. However, the figures for the 2 mg chrysotile and the 5 mg crocidolite clouds were extremely close, 390 and 430 fibres/ml respectively Once again the animals treated with chrysotile had developed significantly more lung fibrosis than those treated with crocidolite. All these results could be taken to indicate that chrysotile is much more fibrogenic than either of the amphiboles, and they might be considered to agree with *in vitro* cytotoxicity studies which have reported that chrysotile causes greater cell damage than either amosite or crocidolite (Klosterkötter and Robock, 1975). From this it might be suggested that the standards for chrysotile should be more strict than for either amosite or crocidolite. In fact, however, consideration of the fibre length distribution of the various dust clouds given in Fig. 1 suggests another possibility. Fibre counting for monitoring the dust clouds supposed to have equal fibre numbers was undertaken using the procedure laid down for the present hygiene standards (Health and Safety Executive, 1976) which records all fibres over 5 µm in length but does not allow for fibre variations above this length. A complete fibre-length distribution produced by scanning electron microscopy showed that the chrysotile clouds used in the present study had many more fibres over 20 µm in length than either of the amphibole clouds (Fig. 1 and 2). No reliable estimates are available relating fibrogenic potential to fibre length, but a number of authors have suggested that for mesothelioma production at least, carcinogenicity depends on fibre lengths in excess of 10 to 20 µm (Maroudas, O'Neal and

Stanton, 1973; Stanton *et al.*, 1977). Since in the present study the only malignant lung tumours were produced by chrysotile and this was also by far the most fibrogenic, these results could support the long-fibre theory of carcinogenesis in general, and also indicate that the same parameters are involved in the fibrogenic response. This would indicate that the present protocol for fibre counting is inadequate, and that either the 5 $\mu$m limit for counting should be raised, or counts should be broken down into different fibre-length groups. Since, however, biological knowledge on the exact lengths of fibre that cause damage is still not definite, a suitable compromise might be to retain the present 5 $\mu$m lower limit, but to include an additional count of fibres $>20$ $\mu$m long. It might be found that this latter figure correlates better with epidemiological data for asbestosis and bronchial carcinomas than the 5 $\mu$m counts.

The finding that chrysotile asbestos produced far more lung fibrosis and pulmonary neoplasia than the amphibole asbestos types was not expected from previous animal inhalation experiments. Wagner *et al.*, in a large study published in 1974, had included groups of rats treated for 12 months with 10 mg/m$^3$ clouds of U.I.C.C. samples of Rhodesian chrysotile, amosite and crocidolite, so that the results should have been directly comparable with those of the present study. However, they reported similar levels of lung fibrosis for all groups, and the number of malignant lung tumours produced by the chrysotile and crocidolite clouds were closely comparable although the amosite cloud produced only one such tumour. The reason for this discrepancy between the 2 studies is difficult to determine, since the dust-retention figures in both studies are extremely close. It may be that the elutriation systems used in the 2 studies differed. Wagner did not give fibre-length distributions for the dust clouds used and it seems likely that the chrysotile clouds used in the present study had a higher proportion of long fibres.

Whether the increased fibrogenic and neoplastic effect of chrysotile found in this study was due to chrysotile itself, or to increased fibre lengths in the chrysotile clouds, it does not change the position regarding human hazards from chrysotile exposure, since the present British industrial asbestos dust standards were based on epidemiological data from chrysotile-exposed working populations. The human position regarding the types of chrysotile cloud met with in industry is, therefore, already known, but the new data indicate that some amphibole clouds may be less dangerous than previously expected.

At present crocidolite is considered in most countries to be the most dangerous asbestos type and its use is banned in some cases. However, this situation is largely due to the association of crocidolite with the production of mesotheliomas in humans. This connection is well documented, but there are no epidemiological data indicating that crocidolite is worse than the other asbestos types at producing lung fibrosis or bronchial carcinomas. The present study would indicate that as far as lung pathology is concerned crocidolite is the least dangerous of the absestos types examined, even though as much as 6 $\times$ more crocidolite than chrysotile was retained after one year of dusting. Because mesothelioma production in response to asbestos inhalation is a very rare event in both animals and humans, animal studies so far undertaken have been unable to produce statistically significant numbers of these tumours for an accurate comparison between the various forms of asbestos. Wagner et al. (1974) reported 5 mesotheliomas from 76 animals with tumours in both the chrysotile and crocidolite groups. We found only 2 mesotheliomas in 123 animals but again both crocidolite and chrysotile were implicated. No mesotheliomas were produced by amosite in either study after 12 months, but Wagner did find one mesothelioma in an animal treated for only one day with amosite dust.

The use of asbestos clouds of differing density over a long inhalation period of 12

months has made it possible to continue the study of asbestos deposition and retention that was commenced using short-term administration of asbestos dust (Middleton *et al.*, 1977). It has been confirmed that the percentage lung retention of chrysotile is much lower than either amphibole types and also that retention is reduced when the density of the cloud is increased. With a 2 mg/m$^3$ cloud the percentage retention of chrysotile is almost double that for a 10 mg/m$^3$ cloud. The retention of crocidolite, however, shows the reverse, and retention is marginally higher with the denser dust cloud. The reasons for this have not been determined with certainty, but measurements of fibre-length distribution of retained lung dust in rats is in progress. These results may indicate the reasons for these differences.

This work was undertaken as part of the research programme funded by the British Asbestos Research Council.

### REFERENCES

ASBESTOSIS RESEARCH COUNCIL (1971) The Measurement of Airborne Asbestos Dust by the Membrane Filter Method. Rochdale (Lancashire). *A.R.C. Technical Note No.* 1.

BECKETT, S. T. (1973) The Evaluation of Airborne Asbestos Using a Scanning Electron Microscope. *Annals Occupat. Hyg.*, **16**, 405.

BECKETT, S. T. (1975) The Generation and Evaluation of U.I.C.C. Asbestos Clouds in Animal Exposure Chambers. *Ann. Occupat. Hyg.*, **18**, 187.

DAVIS, J. M. G. (1974) Histogenesis and Fine Structure of Peritoneal Tumours Produced in Animals by Injections of Asbestos. *J. natn Cancer Inst.*, **52**, 1823.

DEPARTMENT OF EMPLOYMENT AND PRODUCTIVITY (1970) H.M. Factory Inspectorate Standards for Asbestos Dust Concentrations for Use with the Asbestos Regulations, 1969. *DEP. Technical Data Note* 13. London: H.M. Stationery Office.

DOLL, R. (1955) Mortality from Lung Cancer in Asbestos Workers. *Br. J. ind. Med.*, **12**, 81.

DUNMORE, J. H., HAMILTON, R. J. & SMITH, D. S. C. (1964) An Instrument for the Sampling of Respirable Dust for Subsequent Gravimetric Assessment. *J. scient. Instrum.*, **41**, 669.

GLEIT, C. E. & HOLLAND, W. D. (1962) Use of Electrically Excited Oxygen for the Low Temperature Decomposition of Organic Substances. *Anal. Chem.*, **34**, 1454.

GROSS, P. & DE TREVILLE, R. T. P. (1967) Experimental Asbestosis. *Arch. Env. Health*, **15**, 638.

HEALTH AND SAFETY EXECUTIVE (1976) Asbestos Hygiene Standards and Measurement of Airborne Dust Concentrations. *H.S.E. Guidance Note, Environmental Hygiene* 110. London: H.M. Stationery Office.

HOLT, P. F., MILLS, J. & YOUNG, D. K. (1965) Experimental Asbestosis with Four Types of Fibers. *Ann. N.Y. Acad. Sci.*, **132**, 87.

I.A.R.C. (1973) Report of the Advisory Committee on Asbestos Cancers to the Director of the International Agency for Research on Cancer. In *Biological Effects of Asbestos, I.A.R.C. Scientific Publication No.* 8, p. 346.

JACOBSEN, M., RAE, S., WALTON, W. H. & ROGAN, J. M. (1970) New Dust Standards for British Coalmines. *Nature*, **227**, 445.

KLOSTERKÖTTER, W. & ROBOCK, K. (1975) New Aspects on Dust and Pneumoconiosis Research. *Am. indust. Hyg. Ass. J.*, **36**, 659.

MCDONALD, J. C. (1973) Cancer in Chrysotile Mines and Mills. *Biological effects of asbestos, I.A.R.C. Scientific Publication No.* 8, p. 189.

MAROUDAS, N., O'NEAL, C. & STANTON, M. (1973) Fibroblast Anchorage in Carcinogenesis by Fibres. *Lancet*, i, 807.

MIDDLETON, A. P., BECKETT, S. T. & DAVIS, J. M. G. (1977) A Study of the Short-term Retention and Clearance of Inhaled Asbestos by Rats, using U.I.C.C. Standard Reference Samples. In *Inhaled Particles IV* Ed. W. H. Walton. London: Pergamon Press. p. 247.

MORRIS, T. G., ROBERTS, W. H., SILVERTON, R. E & WAGNER, J. C. (1967) In *Inhaled Particles and Vapours*. II. Ed. C. N. Davies. London: Pergamon Press, p. 205.

NATIONAL HEALTH AND MEDICAL RESEARCH COUNCIL (1976) *Membrane Filter Method for Estimating Airborne Asbestos Dust.* Canberra (Australia): N.H.M.R.C.

REEVES, A. L., PURO, H. E. & SMITH, R. G. (1974) Inhalation Carcinogenesis from Various Forms of Asbestos. *Environ. Res.*, **8**, 178.

REEVES, A. L., PURO, H. E., SMITH, R. G. & VORWALD, A. J. (1971) Experimental Asbestos Carcinogenesis. *Environ. Res.*, **4**, 496.

SELIKOFF, I. J., HAMMOND, E. C. & SEIDMAN, H. (1973) Cancer Risk of Insulation Workers in the United States. In *Biological Effects of Asbestos. I.A.R.C. Scient. Publ. No.* 8, p. 209.

STANTON, M. F., LAYARD, M., TEGERIS, A., MILLER, E., MAY, M. & KENT, E. (1977) Carcinogenicity of Fibrous Glass. *J. natn Cancer Inst.*, **58**, 587.

STELL, P. M. & MCGILL, T. (1973) Asbestos and Cancer of Head and Neck. *Lancet*, i, 678.

TIMBRELL, V., HYETT, A. W. & SKIDMORE, J. M. (1968) A Simple Dispenser for Generating Dust Clouds from Standard Reference Samples of Asbestos. *Ann. of Occupat. Hyg.*, **11**, 273.

TIMBRELL, V., SKIDMORE, J. W., HYETT, A. W. & WAGNER, J. C. (1970) Exposure Chambers for Inhalation Experiments with Standard Reference Samples of Asbestos of the International Union against Cancer (U.I.C.C.). *J. Aerosol Sci.*, **1**, 215.

WAGNER, J. C. (1963) Asbestos in Experimental Animals. *Br. J. ind. Med.*, **20**, 1.

WAGNER, J. C., BERRY, G., SKIDMORE, J. W. & TIMBRELL, V. (1974) The Effects of the Inhalation of Asbestos in Rats. *Br. J. Cancer*, **29**, 252.

WAGNER, J. C. & SKIDMORE, J. W. (1965) Asbestos Dust Deposition and Retention in Rats. *Ann. N.Y. Acad. Sci.*, **132**, 77.

WAGNER, J. C., SLEGGS, C. A. & MARCHAND, P.

(1960) Diffuse Pleural Mesotheliomata and Asbestos Exposure in the North Western Cape Province. *Br. J. ind. Med.*, **17**, 260.

WALTON, W. H. & BECKETT, S. T. (1977) A Microscope Eyepiece Graticule for the Evaluation of Fibrous Dust. *Ann. Occupat. Hyg.*, **20**.

ZIELHUIS, R. L. (1977) *Public Health Risks of Exposure to Asbestos.* Report of a Working Group of Experts prepared for the Commission of the European Communities, Directorate-general for Social Affairs, Health and Safety Directorate. Oxford: Pergamon Press.